# Rivastigmine Alleviates Experimentally Induced Colitis in Mice and Rats by Acting at Central and Peripheral Sites to Modulate Immune Responses

**DOI:** 10.1371/journal.pone.0057668

**Published:** 2013-02-28

**Authors:** Helena Shifrin, Mirela Nadler-Milbauer, Shai Shoham, Marta Weinstock

**Affiliations:** Department of Pharmacology, Institute of Drug Research, Hebrew University Medical Center, Jerusalem, Israel; The University of Colorado, United States of America

## Abstract

The cholinergic anti-inflammatory system and α7 nicotinic receptors in macrophages have been proposed to play a role in neuroimmunomodulation and in the etiology of ulcerative colitis. We investigated the ability of a cholinesterase (ChE) inhibitor rivastigmine, to improve the pathology of ulcerative colitis by increasing the concentration of extracellular acetylcholine in the brain and periphery. In combination with carbachol (10 µM), rivastigmine (1 µM) significantly decreased the release of nitric oxide, TNF-α, IL-1β and IL-6 from lipopolysaccharide-activated RAW 264.7 macrophages and this effect was abolished by α7 nicotinic receptor blockade by bungarotoxin. Rivastigmine (1 mg/kg) but not (0.5 mg/kg), injected subcutaneously once daily in BALB/c mice with colitis induced by 4% dextran sodium sulphate (DSS), reduced the disease activity index (DAI) by 60% and damage to colon structure. Rivastigmine (1 mg/kg) also reduced myeloperoxidase activity and IL-6 by >60%, and the infiltration of CD11b expressing cells by 80%. These effects were accompanied by significantly greater ChE inhibition in cortex, brain stem, plasma and colon than that after 0.5 mg/kg. Co-administration of rivastigmine (1 mg/kg) with the muscarinic antagonist scopolamine significantly increased the number of CD11b expressing cells in the colon but did not change DAI compared to those treated with rivastigmine alone. Rivastigmine 1 and 2 mg given rectally to rats with colitis induced by rectal administration of 30 mg dintrobezene sulfonic acid (DNBS) also caused a dose related reduction in ChE activity in blood and colon, the number of ulcers and area of ulceration, levels of TNF-α and in MPO activity. The study revealed that the ChE inhibitor rivastigmine is able to reduce gastro-intestinal inflammation by actions at various sites at which it preserves ACh. These include ACh released from vagal nerve endings that activates alpha7 nicotinic receptors on circulating macrophages and in brainstem neurons.

## Introduction

Inflammatory bowel disease (IBD) comprised of ulcerative colitis (UC) and Crohn’s disease (CD) is a chronic inflammatory disorder of the intestinal tract characterized by genetic, immune and environmental factors contributing to its pathogenesis [1], [2]. Although CD and UC are immunologically different diseases, the chronic inflammation results from dysregulation of the mucosal immune system and of the barrier function of the intestinal epithelium [3], [4]. IBD is characterized by strong macrophage infiltration into the intestinal tissues, the release of pro-inflammatory cytokines and enzymes and the formation of reactive oxygen species. Experimental models have been established in mice and rats that represent several of the pathophysiological features of human UC and CD. A mouse model of UC induced by oral administration of dextran sodium sulphate (DSS) mimics the weight loss, diarrhea accompanied by blood and/or mucus, shortened colon, crypt abnormalities, gastric dysmotility and infiltration of inflammatory cells [5], [6]. A model of hapten-induced Crohn’s disease in rats produced by rectal administration of tri- or dinitrobenzene sulphonic acid (DNBS) is characterized by transmural inflammation, ulceration, and fibrosis [7].

About a third of subjects with UC show impaired regulation of the parasympathetic nervous system. Activation of the efferent vagus by pro-inflammatory cytokines like IL-1β and TNF-α released from macrophages has been shown to attenuate inflammation in a mouse model of acute colitis [8] and in other cytokine mediated inflammatory conditions [9], [10]. ACh released from vagal nerve terminals may interact with α7-nAChR on macrophages or dendritic cells [11] as part of the cholinergic inflammatory pathway first described by Tracey and his colleagues [9], [12], and the activity of this pathway may be deficient in UC. Pro-inflammatory cytokines can also reach the brain via the area postrema (AP) which lies in close proximity to the nucleus of the solitary tract and the dorsomotor nucleus of the vagus (DMN) in the brainstem [13]. The production of pro-inflammatory cytokines in isolated macrophages was shown to be inhibited by acetylcholine (ACh) via activation of nicotinic receptors [9], [14]. Subsequent studies showed that the α7 homopentamer form of the nicotinic receptor (nAChR) was the mediator of the anti-inflammatory effect [15]–[17]. This observation led to the examination of two selective α7 nicotinic agonists, AR-R17779 and GSK1345038A and of nicotine itself in the DSS induced model of colitis in mice [18]. Contrary to expectation, the study revealed that both agonists exacerbated the effects of DSS-induced colitis, while nicotine failed to affect disease parameters although it reduced colonic cytokine production.

Cholinesterase inhibitors (ChEIs) that increase local concentrations of ACh and penetrate the central nervous system (CNS) may be more effective and less toxic than nicotinic agonists in treating colitis. They can act at several sites to reduce inflammation that include parasympathetic and sympathetic ganglia. They can also preserve ACh released from vagal nerve terminals, enabling it to stimulate α7-nAChR on circulating macrophages. ChEIs could also cause indirect activation of muscarinic receptors in the DMN thereby further increasing vagal activity. Rivastigmine, a ChEI, is currently used for the treatment of Alzheimer’s disease and was previously shown to ameliorate immunological and pathological parameters of experimental autoimmune encephalomyelitis in mice [19]. In the present study, the potential anti-inflammatory effect of rivastigmine was examined in the DSS induced model of acute UC in mice and of Crohn’s disease in rats, induced by intracolonic administration of DNBS.

## Materials and Methods

### Quantification of NO Production and Cytokine Release by RAW 264.7 Macrophages

RAW 264.7 macrophages were purchased from ATCC (Manassas, VA, USA). Cells (10^5^cells/well) were seeded in 48-wells plate (Nunc A/C, Rocklide, Denmark) and incubated overnight in complete DMEM. Cells were stimulated by bacterial LPS from *E. coli* (2.5 µg/ml) obtained from Sigma-Aldrich (St. Louis, MO, USA). Working concentrations of LPS was selected based on preliminary dose-response experiments performed on RAW 264.7 macrophages.

RAW 264.7 cells were incubated overnight in complete DMEM containing 4.5 g/L glucose, 1% v/v L-glutamate, 1% v/v penicillin/streptomycin/amphotericin B, 1% v/v sodium pyruvate, 1% v/v Modified Eagle's Medium (MEM), non-essential amino acids solution and 10% v/v Fetal Calf Serum (FCS) (all the above mentioned were purchased from Biological Industries, Israel). Rivastigmine hydrogen tartrate was a gift from Novartis, Switzerland. A working concentration of rivastigmine was chosen that produces 50% enzyme inhibition after 2 h incubation, the time of maximal ChE inhibitory activity (IC_50,_ 1 uM). Carbamoylcholine chloride (carbachol) was obtained from Sigma-Aldrich, (Schnelldorf, Germany). The concentration of LPS was also selected based on preliminary experiments performed with different doses.

RAW 264.7 cells were co-treated with LPS (2.5 µg/ml) and with rivastigmine (1 µM) alone, carbachol (10 µM and 100 µM) alone, or a mixture of rivastigmine (1 µM) with carbachol (10, or 100 µM). Supernatants were collected 24 h later for determination of nitric oxide (NO) by means of Griess reagent which measures the nitrite content in culture medium [20]. TNF-α and IL-6 were measured by sandwich ELISAs in supernatants collected after 6 h and 20 h respectively by means of a BioLegend ELISA Mouse kit (San Diego, CA, USA). Budesonide (100 nM) (St. Louis, MO, USA), a standard anti-inflammatory agent, was applied to the macrophages as a positive control for comparison with rivastigmine. In order to determine whether the anti-inflammatory effect of carbachol combined with rivastigmine resulted from activation of α7-nAChR, a selective nicotinic antagonist α-bungarotoxin (St. Louis, MO, USA) was added to the cells 15 min prior to the addition of rivastigmine and carbachol.

### Determination of Cytotoxicity Following Treatment of RAW 264.7 Macrophages with Carbachol and Rivastigmine

Tests of cell viability were performed on RAW 264.7 cells by the use of MTT (3-(4.5-dimethyl-2-yl)-2.5-diphenyltetrazolium bromide). Cells were co-treated with the compounds and LPS for 24 h. MTT was then added to the cells to give a final concentration of 0.5 mg/ml and the cells incubated for 1 h. Finally, DMSO (St. Louis, MO, USA) was added to solubilize the formazan salt formed which was measured on a plate reader at an OD at 570 nm. The viability of macrophages was quantified as a percentage of the control reading.

### Acute Colitis Induced by DSS in Mice and DNBS in Rats

BALB/c OlaHsd male mice aged 8–9 weeks were purchased from Harlan (Jerusalem, Israel) and housed in standard cages of up to 5 per cage in the Animal Facility of the Institute for Drug Research. Male rats (Sabra Hebrew University strain) weighing 200–250 g were provided by Harlan and housed 2 per cage with free access to food and water. Rats were fasted for 24 h prior to the induction of colitis but were allowed to free access to water. All experiments were carried out in strict accordance with the recommendations in the Guide for the Care and Use of Laboratory Animals of the National Institutes of Health (NIH publication #85-23, revised 1985) protocol no.MD-0912197-4. The protocols were approved by the Committee on the Ethics of Animal Experiments of the Hebrew University of Jerusalem (Permit Number: MD-09-12197; MD-11-12944-4. Animals were anesthetized with CO_2_ before sacrifice and all efforts were made to minimize their suffering. Acute colitis was induced in mice by administration of 4% DSS solution (M.Wt. = 36,000-50, MP Biomedicals, LLC, Solon, OH, USA) in water for 8 days. The DSS solution was freshly prepared every day. Animals were provided with food and water or DSS solution *ad libitum* during the experiment. Animals were divided into the groups of 10–12 as follows: PBS group: mice drank water and were injected subcutaneously (s.c.) with phosphate buffered saline (PBS) once daily for 8 days; Drug control groups: animals drank water and were injected s.c. with rivastigmine (0.5 mg/kg or 1 mg/kg) once daily for 8 days. DSS untreated group: mice drank 4% DSS and were injected s.c. with PBS once daily for 8 days; Drug treated groups: All mice drank 4% DSS and were injected s.c with rivastigmine (0.5 mg/kg, 1 mg/kg or 2 mg/kg) once daily for 8 days; or rivastigmine s.c. (1 mg/kg) once daily and scopolamine hydrobromide s.c. (1 mg/kg) (Teva, Israel) twice daily. The volume of 4% DSS solution consumed by each group of mice was measured and the average volume/mouse was calculated. On the eighth day, animals were anesthetized by CO_2_, blood samples were collected by cardiac puncture and mice were sacrificed by cervical dislocation.

In rats, colitis was induced under light ether anesthesia by intracolonic administration of 30 mg DNBS, (Sigma, Israel) dissolved in ethanol 30% (v/v). A perforated Foley catheter was inserted rectally for 5–8 cm from the anus and removed immediately after administration of DNBS. The rats were left in an upside down position for additional 30 s. They were divided into 3 groups of up to 13 animals. Rivastigmine (1 and 2 mg/kg) was administered rectally twice daily for three days and the first dose was given 1 h after DNBS. Control rats were given DNBS or distilled water and then distilled water twice daily instead of rivastigmine. On the fourth day, the rats were anesthetized with an intraperitoneal injection of 100 mg/kg body weight of ketamine (Ketaset, USA). Blood samples were taken from vena cava prior to sacrificing the rats by puncturing of the chest wall.

### Cholinesterase Inhibition in Mice and Rats

On the 8th day of the experiment, 10 mice in each of the drug-treated and control animals were injected s.c with rivastigmine (0.5 or 1 mg/kg) 50 min (time of peak cholinesterase inhibition) [21] prior to sacrifice by cervical dislocation. The brain was rapidly removed and the frontal cortex and brain stem areas dissected out, immediately frozen in liquid nitrogen and stored at -80°C. Colon segments of about 0.5 cm in length were taken from the proximal area, washed in cold PBS, frozen in liquid nitrogen and stored at −80°C. Mouse blood taken by cardiac puncture was added to heparin-containing (Trima, Maabarot, Israel) Eppendorf tubes and centrifuged at 4°C 13,000 rpm for 3 min. The plasma was frozen in liquid nitrogen and stored at −80°C. The same procedure was applied to the rats’ blood taken from the vena cava. Colon tissue from mice and rats was homogenized in phosphate buffer 0.03 M, pH containing 1% Triton-100 (Baker, Phillipsburg, NJ, USA), centrifuged at 4°C for 15 min at 14000 rpm and the supernatants were taken for the analysis by the method of Ellman et al, [22]. ChE activity was expressed as µM of acetylthiocholine hydrolyzed/min/mg protein measured in the sample and protein determined by means of a BCA Protein Assay kit (Thermo Scientific, USA) according to manufacture instructions. Inhibition of ChE in drug-treated groups was expressed as percent reduction in activity of that in the respective DSS or DNBS treated groups injected s.c. with saline or with distilled water administered rectally.

### Evaluation of Clinical Signs of Acute Colitis in Mice

Body weight, stool consistency and the presence of blood in the stools was recorded daily for each mouse. The data were used to calculate a disease activity index (DAI) as described by Kullmann et al, [23]. The score ranged between 0–4 for all three parameters and was composed as follows: Weight loss: none = 0, 1–5% = 1, 5–10% = 2, 10–20% = 3, >20% = 4. Percent of weight loss at the end of the experiment relative to the first day was calculated for all the groups. Stool evaluation: normal pellets = 0, loose stools which do not stick to the anus = 2, diarrhea = 4. Bleeding: none = 0, hemoccult = 2, gross bleeding = 4. The maximum DAI score was 4 based on combination of scores of weight loss (refer to the first day of the experiment), stool consistency and bleeding divided by 3. After sacrifice the length of the colon segment from the cecum to the rectum was measured.

### Evaluation of Signs of Acute Colitis in Rats

The distal part of rats’ colon was removed, open and rinsed with ice-cold PBS, pH 7.4. Colon sections were blotted dry, weighed and examined for ulcerated and inflamed regions. The number of ulcers was counted and their area was measured and colon sections were frozen in liquid nitrogen and stored at −80°C.

### Cytokine Detection in Colonic Tissues of Mice and Rats

TNF-α, IL-6, and IL-1β levels in homogenized colon were measured by means of a BioLegend ELISA Mouse kit. One cm of colon was homogenized in ice-cold PBS buffer containing protease inhibitor cocktail (Sigma, St. Louis, MO, USA; dilution 1∶100) using a Polytron homogenizer, and then centrifuged for 15 min at 14,000 rpm at 4°C. Supernatants were diluted 1∶5 in diluent buffer supplied in the kit and applied to 96-well MaxiSorb Elisa plates according to manufacturer’s protocol. Total protein concentration in the tissue supernatant was measured by means of a BCA kit. Levels of cytokines are expressed as pg/mg total tissue protein.

### Biochemical Analyses in Colonic Tissues of Mice and Rats

Neutrophil infiltration was monitored by measuring myeloperoxidase (MPO) activity in colon segments according to the protocol described by Bradley [24] in 96-well micro-titer plates by mixing 10 µl of the supernatant with 290 µl of o-dianisidine dihydrochloride solution. Data were interpolated from a MPO standard curve. MPO activity was expressed as units per mg total protein measured as described above. Lipid peroxidation (TBARS) was determined in rat colon samples according to the procedure previously described in [25].

### Reverse Transcription and Quantitative Real-time Polymerase Chain Reaction (RT-PCR) Performed on Mouse Colon Sections

A small segment (0.5–0.7 cm) of the distal colon was removed from each mouse, snap frozen in liquid nitrogen and stored at −80°C. Colon samples were homogenized by a Polytron homogenizer (Kinematika AG, Switzerland) and then extracted with a Total RNA mini kit (Genaid Biotech, Sijhih, Taiwan) according to manufacturer’s instructions for tissue. RNA integrity was verified by means of electrophoresis in denaturing gels. Gels were stained with ethidium bromide (Amresco, OH, USA) and the ratio of 28S to 18S was examined visually. RNA concentration was assessed by measuring the absorbance at 260 nm using NanoDrop machinery. One microgram of RNA from each sample was reverse transcribed using High Capacity cDNA Reverse Transcription Kit (Applied Biosystems, Carlsbad, CA, USA) according to the manufacturer's instructions. The RT reaction was run for 10 min at 25°C, 2 h at 37°C and 5 min at 85°C. Relative levels of mRNA were examined using TaqMan chemistry with appropriate TaqMan probes. Real time PCR reactions were performed in 96-well plates in the ABI-Prism 7900 Sequence Detector (Applied Biosystems, CA) on fast mode. Each sample was run in triplicate (reaction final volume was 10 µl) at StepOnePlus™ RT-PCR System (Applied Biosystems). TNF-α, IL-6, IL-1β mRNA was measured in samples extracted from mouse colon and normalized to Ubiquitin C mouse gene (UBC, Applied Biosystems, CA).

### Immunohistochemical Analyses in Mice

After sacrifice, the abdominal cavity of the mice was opened and the colon located, removed, sectioned longitudinally and rinsed with sterile ice-cold PBS buffer to remove feces. The whole colon was divided into several segments and the section between distal and proximal part (2.5–3 cm) was excised, fixed in 4% formalin (Sigma, St. Louis, MO, USA), and embedded in paraffin. For histological observations the slides were stained with hematoxylin and eosin. Immunohistochemical analysis of colon segments was performed according to the protocol described by Dmitrieva et al, [26]. Sections were blocked with avidin and biotin from Avidin/Biotin blocking kit (Vector Laboratories, Inc, Burlingame, CA), washed and incubated with primary rat anti mouse CD11b antibody 1∶400 (Merck Millipore, USA) overnight at 4°C. Tissues were rinsed with PBS, incubated with biotinylated sheep anti rat secondary antibody 1∶200 (Boehringer, Germany) for 1 h and stored at 4°C overnight. After rinsing, streptavidin conjugated with Cy3 (Sigma-Aldrich, St. Louis, MO, USA) at dilution 1∶200 was applied for 1 h at room temperature, followed by 4′,6-diamidino-2-phenylindole (DAPI; Molecular Probes, USA) and counterstaining (blue) for 5 min. Finally, samples were washed in PBS and mounted with Immu-Mount medium (Thermo Scientific, USA). Images were processed with ZEISS LSM710 confocal microscope (Carl Zeiss, Oberkochen, Germany).

For quantification of infiltration of CD11b expressing cells three fields measuring 250×250 µm were sampled from each mouse. Using image analysis software (Image J) pink (co-localized) cells that have a range of ovoid and crescent shapes were counted [27].

### Release of TNF-α and IL-6 Protein from Peritoneal Macrophages of DSS Treated Mice

In order to determine whether rivastigmine acts locally by inhibiting ChE in vagal nerve endings, thereby increasing ACh in the circulation and its activation of macrophages, we collected peritoneal cells by lavage of control mice and those given DSS with and without rivastigmine (0.5 or 1 mg/kg) as described in the preceding section. Peritoneal macrophages were cultured with LPS (10 µg/ml) for 6 and 20 h, supernatants were harvested and cytokine production was quantified using ELISA as described above.

### Statistical Analyses

Results are expressed as mean values ± SEM. Statistical analysis of data was performed by ANOVA for group followed by Duncan’s *post hoc* test. All statistical analyses were performed using IBM ® SPSS® 19 software. Differences were considered statistically significant if the p value was <0.05. All doses and concentrations of rivastigmine are expressed in µM or mg/kg of the tartrate salt.

## Results

### Release of Nitric Oxide and Cytokines from LPS-activated RAW 264.7 Cells

**Figure 1:**
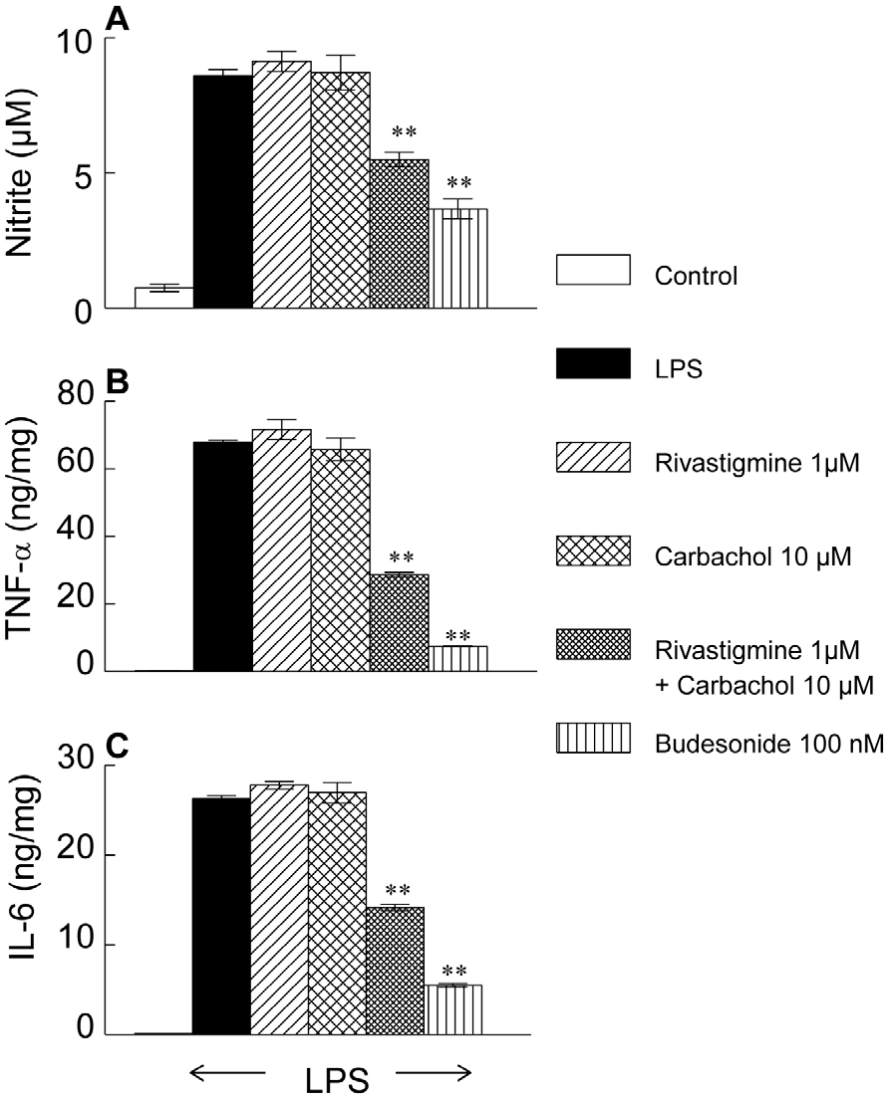
Effect of rivastigmine and carbachol on the release of NO and pro-inflammatory cytokines in macrophages. Data represent the mean ± SEM of 3 independent experiments performed in triplicates. Significantly different from LPS alone, **p<0.01.

LPS increased the release of NO from RAW 264.7 cells about 10-fold (Figure 1A). This was unaffected by rivastigmine (1 µM), or carbachol (10 µM) given separately. A combination of rivastigmine (1 µM) with carbachol (10 µM) reduced NO release by 35% No further reduction was achieved by rivastigmine and carbachol (100 µM). LPS given alone, or together with rivastigmine (1 µM), carbachol (10 µM), or a combination of both drugs, did not have a cytotoxic effect on activated cells, as revealed by MTT assay (data not shown).

**Figure 2:**
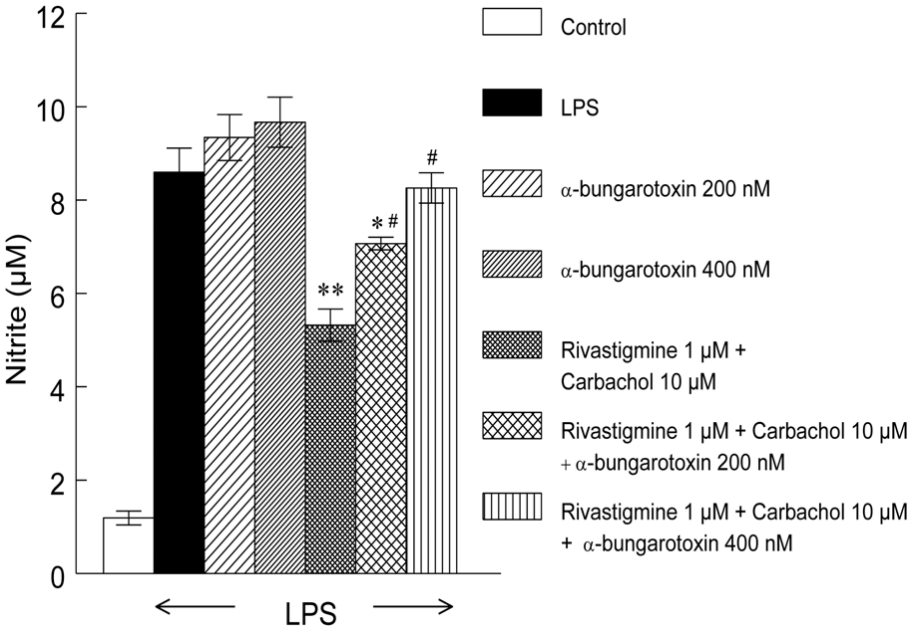
α-bungarotoxin inhibits effect of rivastigmine and carbachol on release of NO in LPS-activated macrophages. Data represents mean ± SEM of 2 independent experiments performed in eight replicates for each sample. Significantly different from LPS alone *p<0.05, **p<0.01; significantly different from macrophages pretreated with rivastigmine+carbachol; # *p*<0.05.

Carbachol (10 µM) and rivastigmine (1 µM) applied individually did not cause any significant reduction in pro-inflammatory cytokines. A combination of rivastigmine (1 µM) with carbachol (10 µM) reduced TNF-α and IL-6 by 50% and 46%, respectively. The steroid budesonide (100 nM) reduced the level TNF-α by 90% and IL-6 by 80% (Figures 1B and C). Pre-incubation with α-bungarotoxin antagonized the effect of rivastigmine plus carbachol in a dose-related manner (Figure 2).

### Effect of Rivastigmine on ChE Activity in Mouse Colon, Brain and Plasma

ChE activity in the colon of mice injected with PBS drinking 4% DSS solution did not differ significantly from that in control mice drinking water and was 30.7±2.1 and 26.1±2.1 µmoles acetylthiocholine hydrolysed/min/g protein, respectively. ChE inhibition in the cortex, brainstem, colon and plasma of mice treated with rivastigmine and drinking 4% DSS solution is shown in Table 1. Rivastigmine (1 mg/kg) inhibited ChE by 57–67% which was significantly greater (*p*<0.01) than that of the lower dose in all tissues examined.

**Table 1:** Cholinesterase inhibition by rivastigmine in colon, plasma, and brain of DSS drinking treated mice.

Group	Cortex	Brain Stem	Colon	Plasma
Rivastigmine 0.5	52.3±1.0	36.9±2.95	48.9±2.8	39.2±4.1
Rivastigmine 1	65.6±1.4**	57.5±1.9**	65.4±4.1**	66.6±2.3**

Rivastigmine 0.5 or 1 = mice treated with rivastigmine (0.5 mg/kg or 1 mg/kg) once daily. Data represent percent ChE inhibition induced by rivastigmine compared to that in mice treated with PBS and drinking DSS. Data represent the mean ± SEM from 10 animals. Significantly different from value in mice treated with rivastigmine (0.5 mg/kg), **p<0.01.

### Effect of Rivastigmine Treatment on Macroscopic Markers of DSS Induced Colitis in Mice

Control mice injected with rivastigmine (0.5 mg/kg) or (1 mg/kg) that drank water for 8 days showed no reduction in their average daily water consumption or signs of diarrhea or bleeding. However, treatment of such mice with rivastigmine (1 mg/kg) caused a 4.7% reduction in body weight at the end of the experiment. No significant difference was detected in the consumption of 4% DSS solution among the various groups of mice. Diarrhea with occult blood was the earliest clinical sign of acute colitis and appeared on the 5^th^ day but there were no significant differences in the DAI score for any of the groups drinking DSS solution with or without treatment. On day 8 of DSS treatment signs of colitis were more severe and included loose stools or diarrhea, blood in feces and weight loss of more than 5% (Table 2). Macroscopic examination in mice injected with PBS revealed a significant decrease of 40% in colon length and signs of hyperemia and inflammation. Rivastigmine (1 mg/kg), but not (0.5 mg/kg), partially antagonized colon shrinkage (Table 2) and completely prevented bleeding. DAI calculated for the group treated with rivastigmine (1 mg/kg), but not (0.5 mg/kg) was significantly different from that of the group drinking DSS given PBS. Treatment with rivastigmine (2 mg/kg) once daily, caused an average weight loss of 15% probably because of reduced food consumption since it caused little diarrhea. Therefore no more experiments were performed with this dose.

**Table 2:** Effect of rivastigmine treatment on macroscopic parameters of acute colitis in mice treated with DSS.

Group	Colon length (cm)	Weight loss (%)	Bleeding (score)	Stool (score)	DAI Day 5	DAI Day 8
Water +						
PBS	9.9±0.1	0	0	0	0	0
Riv 0.5	9.2±0.3	0.7±1.8	0	0	0	0
Riv 1	9.6±0.4	4.7±1.5**	0	0	0.3±0.3	0.3±0.6
DSS +						
PBS	6.0±0.2**	15.1±2.3**	3.1±0.4**	2.8±0.3**	0.9±0.2	2.9±0.2**
Riv 0.5	6.2±0.1	8.0±1.3‡‡	2.9±0.4	3.3±0.2	0.5±0.1	2.8±0.2
Riv 1	7.9±0.3‡‡ ^#^	11.7±1.6	0.4±0.2‡‡ ^##^	0.8±0.3‡‡ ^##^	0.6±0.1	1.2±0.2‡ ^#^
Riv 1+ Scop 1	6.3±0.1§	0§§	0.7±0.5	1.3±0.7	0.6±0.2	0.7±0.3

DDS = dextran sulphate solution. PBS phosphate buffered saline. Riv 0.5 or 1 = mice treated with rivastigmine (0.5 or 1 mg/kg) once daily; Riv 1+ Scop 1 = mice treated with rivastigmine (1 mg/kg) once daily and scopolamine (1 mg/kg) bid; DAI = disease activity index. Significantly different from water+PBS ** p<0.01; significantly different from DSS+PBS,

‡ p<0.05.

‡‡ p<0.01; significantly different from DSS+Riv 0.5 mg,

# p<0.05;

## p<0.01; significantly different from DSS+Riv 1 mg/kg,

§ p<0.05;

§§ p<0.01.

Mice given scopolamine (1 mg/kg) to block muscarinic receptors in the DVN to see if this would reduce the anti-inflammatory effect of rivastigmine (1 mg/kg), exhibited marked hyperactivity and aggressive behavior, resulting in the death of two of them. Nevertheless, in the remainder, scopolamine prevented the protective effect of rivastigmine on colon length but not on bleeding and diarrhea (Table 2).

### Effect of Rivastigmine Treatment on Cytokine Release and MPO Activity in Mouse Colon

**Figure 3:**
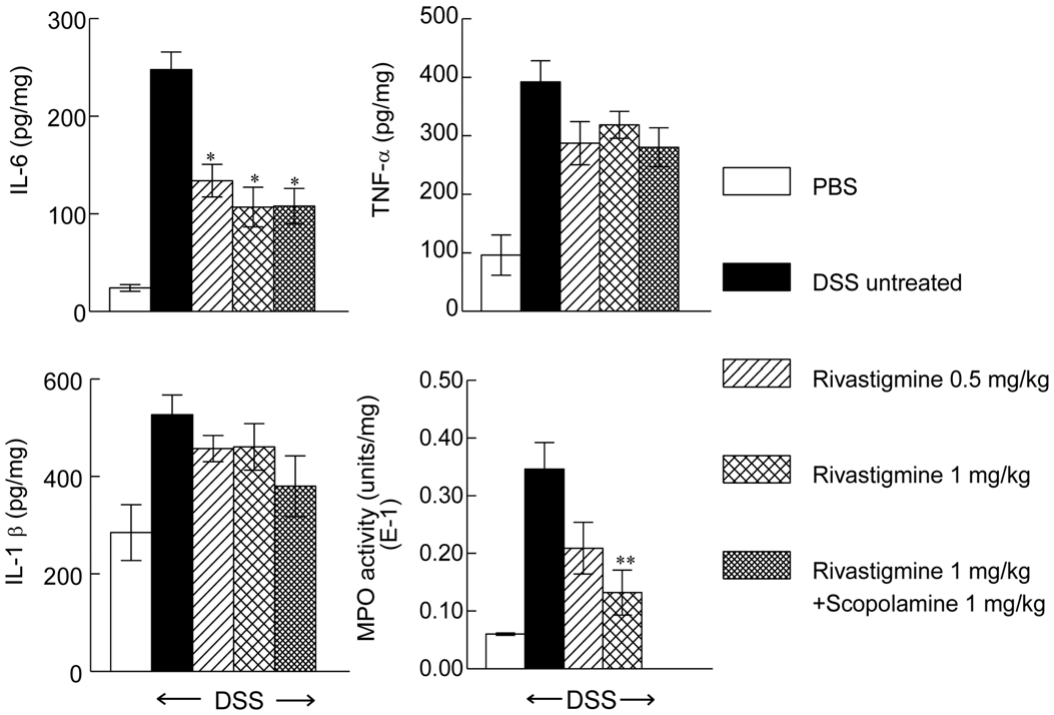
Effect of rivastigmine on cytokines and MPO activity in colon of mice with DSS-induced colitis. PBS = phosphate buffered saline. DSS = dextran sodium sulphate. Data are mean ± SEM, Significantly different from mice treated with PBS and drinking DSS *p<0.05, **p<0.01.

DSS solution causes an abnormal response to luminal flora resulting in the release of a burst of pro-inflammatory cytokines. This was also seen in the current study in which levels of TNF-α, IL-6 and IL-1β protein increased 3-fold, 7-fold and 1.7-fold respectively on the 8th day of drinking DSS solution. Rivastigmine (0.5 mg/kg and 1 mg/kg) reduced by about 50% and 60% respectively, the concentration of IL-6 but not those of TNF-α and IL-1β (Figures 3 A–C). Co-treatment with scopolamine did not affect the reduction in IL-6 induced by rivastigmine (1 mg/kg). There was a 7-fold increase in MPO activity in the colon in mice with DSS-induced colitis on the 8th day of the experiment. Treatment with rivastigmine (0.5 and 1 mg/kg) reduced MPO by 40% and 70%, respectively (Figure 3D). The activity of MPO increased in proportion to the DAI score for all groups (Table 2 and Figure 3D).

**Figure 4:**
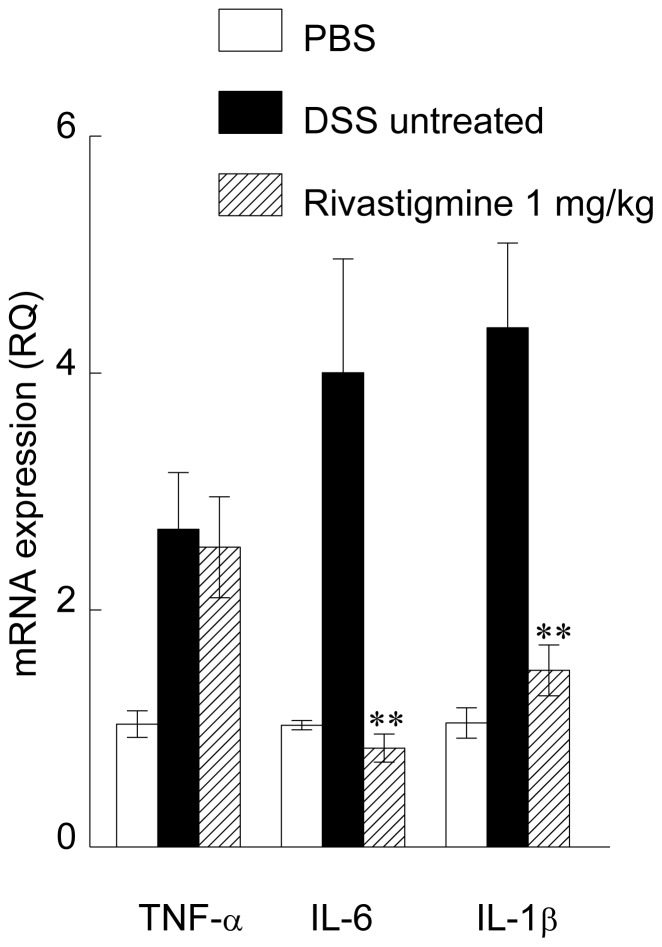
mRNA of pro-inflammatory cytokines in colon of mice with DSS- induced colitis. Legend as in Figure 3. Data are mean ± SEM and represents pooled data from 9–10 mice per group. Significantly different from mice with DSS-induced colitis treated with PBS, ** *p*<0.01.

DSS induced colitis also caused a 2-fold increase in the expression of TNF-α mRNA and 4- and 5- fold increases in the expression of IL-6 and IL-1β, respectively. Treatment with rivastigmine (1 mg/kg) did not affect the expression of TNF-α mRNA, but reduced that of IL-6 and IL-1β by about 70%. The mRNA level of IL-1β was significantly reduced in the rivastigmine treated group in contrast to protein measurements in total colon homogenates (Figure 4).

### Histopathological Characterization of Mouse Colon

**Figure 5:**
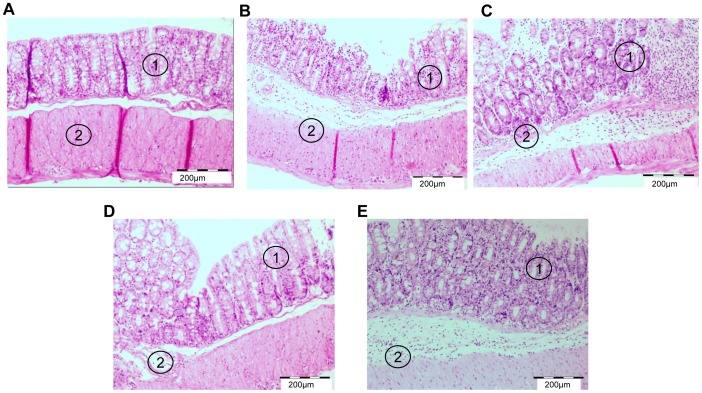
Histopathological characterization of colon segments mice with DSS-induced colitis. Magnification×10. A: Distal colon sections of control mouse showing well organized crypts with goblet cells (1) and lamina propria (2). B: Colon of PBS-injected mouse with DSS-induced colitis showing crypt loss (1), mucosal erosions, sub-mucosal edema and massive infiltration of inflammatory cells (2). C: Colon of mouse treated with rivastigmine (0.5 mg/kg) that partially repaired the crypt dysplasia (1) but had little effect on cellular infiltrate and edema of the sub-mucosal layer (2). D: Colon of mouse treated with rivastigmine (1 mg/kg) that partially restored crypt damage (1), reduced sub-mucosal edema (2) and prevented cellular inflammatory infiltrate. E: Co administration of scopolamine (1 mg/kg) and rivastigmine (1 mg/kg) increased sub-mucosal edema and cellular infiltrate compared to that in colon of mice given rivastigmine (1 mg/kg) alone.

The colon of mice drinking DDW and injected with PBS revealed well organized crypts and an intact sub-mucosal layer and lamina propria (Figure 5A). Mice with DSS-induced colitis injected with PBS showed structural damage to the colon with erosions of the crypts and sub-mucosal edema. Inflammation involved all layers of the colon with massive infiltrates discernible in the lamina propria (Figure 5B). Treatment with rivastigmine (0.5 mg/kg) caused little change in these pathological manifestations (Figure 5C), but rivastigmine (1 mg/kg) caused a partial restoration of the structure of the crypts and a reduction in sub-mucosal edema and cell infiltration (Figure 5D). Co-administration of scopolamine (1 mg/kg) with rivastigmine (1 mg/kg) increased sub-mucosal edema and cellular infiltrates compared to those given rivastigmine alone (Figure 5E).

### Effect of Rivastigmine Treatment on Infiltration of CD11b+ Cells

**Figure 6:**
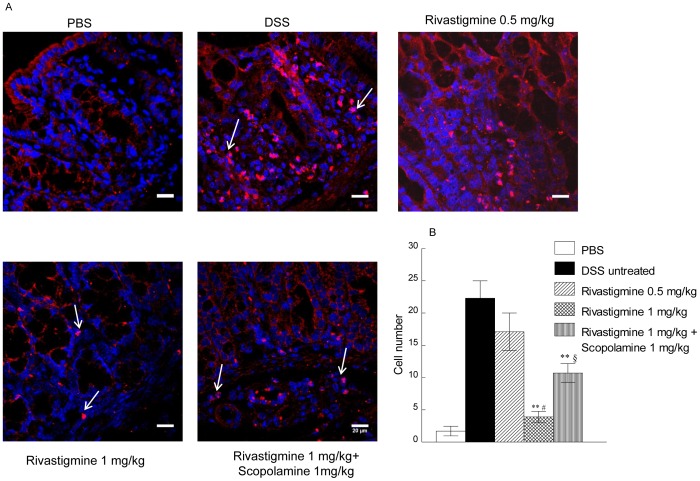
Effect of rivastigmine with and without scopolamine on CD11b+ cell infiltration in DSS-induced colitis. A: Immunofluorescence stained mouse tissue sections showing CD11b (red) cells. White arrows show cells which express with CD11b+ in colitis tissue (pink). B: Quantification of the pink CD11b+ expressing cells (ImageJ) in the samples of mice colon sections. Data represent the mean ± SEM from three slides per animal, 6 animals per group; scale bar 20 µm. Significantly different from mice with DSS-induced colitis treated with PBS, **p<0.01; significantly different from mice treated with rivastigmine (0.5 mg/kg), # *p*<0.05; significantly different from mice treated with rivastigmine (1 mg/kg), ^§^ *p*<0.05.

In mice treated with PBS and 4% DSS there was a significant infiltration of CD11b expressing cells (Figure 6A). Treatment with rivastigmine (1 mg/kg) but not (0.5 mg/kg) reduced the number of CD11b expressing cells by more than 80% (Figure 6B). Scopolamine given together with rivastigmine (1 mg/kg) significantly increased the number of CD11b expressing cells in the colon compared to those given rivastigmine alone (*p*<0.05).

### Reduction by Rivastigmine of TNF-α Release from Peritoneal Macrophages of Mice with DSS-induced Colitis

**Figure 7:**
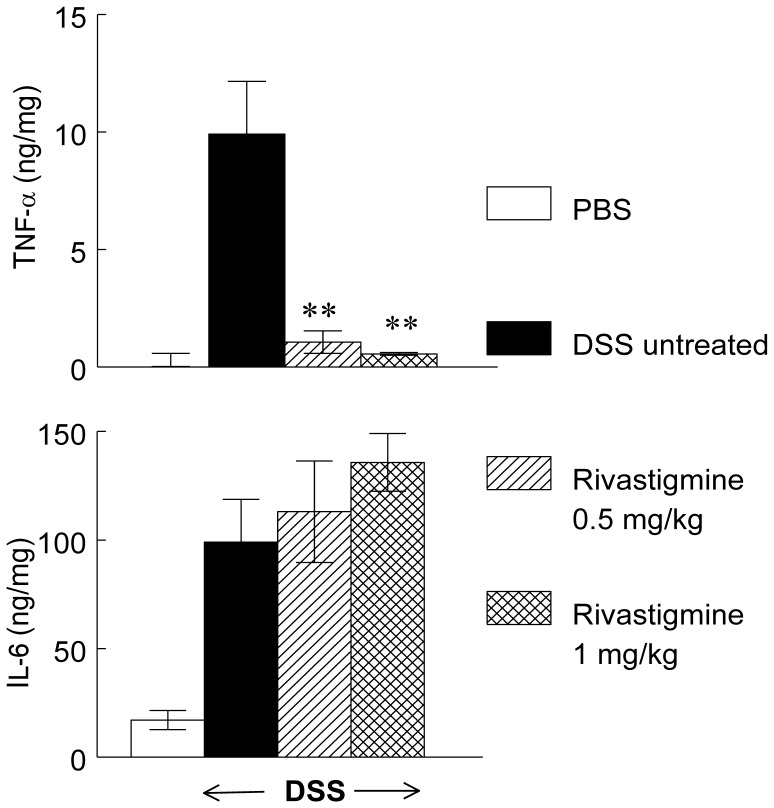
Effect of rivastigmine on TNF-α and IL-6 released from peritoneal macrophages of DSS treated mice. Legend as in Figure 3. Data are mean ± SEM and represent pooled data from 6 animals per group. Significantly different from mice with DSS-induced colitis treated with PBS, ** *p*<0.01.

Levels of TNF-α and IL-6 proteins were very low or undetectable in un-stimulated peritoneal macrophages extracted from PBS treated and rivastigmine-treated mice. However, when stimulated by LPS (10 µg/ml) for 6 h (TNF-α) and 20 h (IL-6) the release of these cytokines increased about 10-fold. In contrast to our findings in the colon, rivastigmine (0.5 or 1 mg/kg) caused a significant reduction in TNF-α but not of IL-6 in peritoneal macrophages compared to those injected with PBS (Figure 7).

### Effect of Rivastigmine Treatment on Macroscopic Markers of DNBS Induced Colitis in Rats

The small weight gain of 27 g seen on the 4th day in rats given distilled water rectally did not occur in those treated with DNBS with and without rivastigmine. This resulted in a significant increase in the ratio of colon to body weight in DNBS treated rats since there was some colonic swelling induced by DNBS, which was not prevented by administration of rivastigmine (1 or 2 mg/kg). However, both doses of rivastigmine significantly reduced the number of ulcers/rat (*p*<0.01) while the higher dose also reduced the area of ulceration (*p*<0.01) (Table 3).

**Table 3:** Effect of rivastigmine treatment on macroscopic parameters of acute colitis induced in rats by DNBS.

Group	Change in body weight (%)	Colon weight/body weight (%)	Number of ulcers/rat	Area of ulcers (cm^2^)
DW	8.3±0.7**	0.40±0.02**	0**	0**
DNBS	1.1±1.4	0.63±0.03	2.27±0.42	1.79±0.49
DNBS+ Riv 1 (mg/kg)	−1.1±1.5	0.61±0.08	0.77±0.41**	1.04±0.55
DNBS+ Riv 2 (mg/kg)	−1.1±1.6	0.57±0.05	0.31±0.25**	0.04±0.04**

DNBS = dinitrobenzene sulphonic acid. DW = distilled water. Riv = rivastigmine. Significantly different from DNBS, **p<0.01.

### Effect of Rivastigmine Treatment on Cytokine Release, MPO Activity TBARS, TNF-α and ChE Activity in Rat Colon

**Figure 8:**
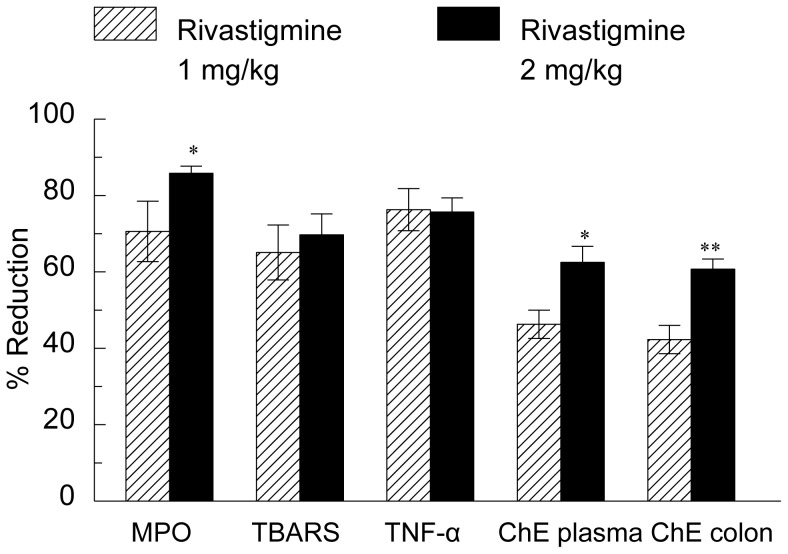
Effect of rivastigmine on TNF-α, MPO, TBARS and ChE activity in rats with DNBS-induced colitis. ChE = ChE activity. Data are mean ± SEM from 13 animals per group. Percent of reduction compared to values in group treated with distilled water and DNBS. Significantly different from the rats treated by rectal administration of rivastigmine (1 mg/kg) *p<0.05, ** *p*<0.01.

There were no significant differences in the activity of ChE in the colon of rats drinking water (41±10.3) or those given DNBS (32.9±6.0 µmoles acetylthiocholine hydrolysed/min/gm protein) or in plasma (5.92±0.42 and 5.88±0.48 µmoles acetylthiocholine hydrolysed/min/gm protein), respectively. MPO activity in the colon of rats treated with DNBS increased almost 10-fold to 54±10 from 5.7 U/mg protein in controls drinking water, while TBARS increased from 0.78±0.07 µM/mg protein in control rats to 2.01±0.26 µM/mg protein in rats treated with DNBS. TNF-α in the colon increased from 0.035±0.01 ng/mg protein in control rats to 0.168±0.02 ng/mg protein in those treated with DNBS. The effect of rivastigmine on ChE activity in the colon and plasma and on colonic TNF-α, MPO activity, and TBARS was calculated as per cent change of that in rats treated with DNBS and is shown in Figure 8. Rivastigmine (1 mg/kg) decreased MPO activity, TBARS and TNF-α in the colon by more than 60%, colonic and plasma ChE by 40–45%. Rivastigmine (2 mg/kg) caused a significantly greater reduction in MPO and ChE activity in colon and plasma than a dose of 1 mg/kg.

## Discussion

Tracey and his colleagues described a cholinergic anti-inflammatory system in rodents and suggested that it may provide the link in neuroimmunomodulation [9], [12], [13], [28]. This anti-inflammatory system comprises the α7 subunit of the nAChR on circulating macrophages that are believed to be stimulated by ACh released from efferent vagal nerve terminals in response to activation of the afferent vagus by pro-inflammatory cytokines. Cytokines also have direct access to the brain stem via the AP [13] from which they can cause the activation of the efferent vagus and sympathetic nervous system to release ACh, noradrenaline and adrenaline.

After interacting with the α7-nAChR, ACh can affect cytokine release by reducing the transcription factor NF-κB [29], [30] and by inhibiting the Jak2/STAT3 pathway [31]. Recent studies have shown that the spleen is a major source of inflammatory cytokines involved in the initiation of systemic inflammation [32]. ACh can activate postsynaptic α7-nACh receptors in splenic nerve ganglia increasing the release of noradrenaline in the spleen thereby inhibiting cytokine production [33].

The current study examined the potential of a ChEI rivastigmine as treatment for colitis by using the DSS model in mice and DNBS in rats. We first showed that rivastigmine can potentiate the anti-inflammatory effect of the cholinergic agonist carbachol, mediated via the α7-nAChR on a macrophage cell line. It was necessary to give carbachol (which is hydrolyzed by AChE, albeit more slowly than ACh) with the ChEI since there is no evidence that macrophages can synthesize ACh, although they express AChE mRNA and nAChR [11]. The combination of rivastigmine and carbachol decreased the release of NO and pro-inflammatory cytokines, TNF-α and IL-6, from the macrophages after their activation by LPS and this effect was antagonized by α-bungarotoxin, testifying to the involvement of the α7-nAChR in its mediation. Treatment of mice with DSS-induced colitis with rivastigmine (1 mg/kg) for 8 days almost completely prevented the release of TNF-α from macrophages harvested from the mice and activated by LPS. This finding indicated that preservation by rivastigmine of ACh released from vagal nerve terminals and in relevant brain neurons can bring about the activation of α7-nAChR in circulating macrophages and inhibit LPS-induced cytokine release.

In the DSS model of colitis, we examined the effect of two doses of rivastigmine (0.5 or 1 mg/kg), administered once daily by s.c. injection. Rivastigmine (1 mg/kg) inhibited ChE in the colon, plasma, cerebral cortex and brainstem to a significantly greater extent than 0.5 mg/kg. In spite of the fact that both doses decreased the production of IL-6 in the colon by more than 50%, only the higher dose significantly reduced the macroscopic and microscopic parameters of colitis. The lack of effect of rivastigmine (0.5 mg/kg) is reminiscent of that which was reported for nicotine (2.5 µmoles/kg) which also decreased colonic IL-6 by about 50% but failed to affect crypt loss, mucosal erosions, sub-mucosal edema and infiltration of inflammatory cells. Although DSS increased both IL-1β and TNF-α in the colon neither was significantly reduced by rivastigmine, just as reported for nicotine [18]. Rivastigmine (0.5 mg/kg) reduced MPO in the colon by about 40% but this did not quite reach statistical significance. Neither did this dose reduce the number of activated macrophages in the colon. By contrast, rivastigmine (1 mg/kg) virtually abolished rectal bleeding, decreased the diarrhea score by >50% and increased colon length by 30%. It also partially restored the damage to crypt structure caused by DSS, reduced sub-mucosal edema and colonic MPO activity by more than 60% and prevented the infiltration of inflammatory cells. Thus, reduction of IL-6 alone in the colon is insufficient to confer protection against the clinical signs of colitis unless it is accompanied by a substantial reduction in the infiltration of the colon by activated macrophages.

The comparative data obtained with the two doses of rivastigmine suggest that the degree of ChE inhibition in plasma, colon or brain is critical to obtain an optimal therapeutic effect of the drug in this acute model of colitis. ChE inhibitors galantamine and huperzine, reduced TNF-α in the serum and spleen and improved survival of mice with endotoxemia induced by injection of LPS [28]. In contrast to rivastigmine, which binds covalently to ChE, inhibition of this enzyme by these reversible ChEIs cannot be measured directly *ex vivo* [34]. The improvement of mouse survival by galantamine was prevented by co administration of atropine but not atropine methyl nitrate that does not penetrate the CNS [28]. This indicated that the anti-inflammatory effect of galantamine was mediated by indirect activation of muscarinic receptors resulting from ChE inhibition in the brain. The role of muscarinic M1 receptors in the brain was established by the finding that intracerebroventricular injection of selective muscarinic M1 agonists dose-dependently reduced serum levels of TNF-α in endotoxin injected rats [35]. It was also shown that stimulation of α7-nAChR in macrophages was an essential component of its anti-inflammatory effect since it was not seen α7-nAChR knock-out mice. In another study in Sprague-Dawley rats with colitis induced by DNBS, ChE inhibitors neostigmine and physostigmine reduced macroscopic damage and MPO activity in the colon. Although no measurements were made of ChE activity, it was shown that physostigmine had a greater effect than neostigmine on the parameters of colitis [36]. Since neostigmine has a quaternary N and does not penetrate the CNS, the authors concluded that stimulation of central cholinergic receptors contributed to the greater anti-inflammatory effect of physostigmine.

In the current experiment we were able to show that activation of central M1 receptors contribute to the anti-inflammatory of the latter in colitis since the effect of rivastigmine was reduced by co-administration of scopolamine, a centrally acting muscarinic antagonist. Confirmation that scopolamine acted on the CNS was seen in the characteristic hyperactivity induced in the mice. Scopolamine significantly decreased the protective effect of rivastigmine on the colonic infiltration by CD11b staining cells and sub-mucosal edema and prevented the increase in colon length, but did not affect the reduction in IL-6 in the colon. Scopolamine prevented the reduction in weight loss detected in mice also given rivastigmine (1 mg/kg) possibly by antagonism of an effect of rivastigmine on food intake.

In the rat model of colitis induced by rectal administration of DNBS, rivastigmine caused a dose related reduction in the area of ulceration and number of colonic ulcers and in TBARS (a measure of oxidative stress) colonic MPO and ChE activity. In contrast to the failure of rivastigmine to reduce TNF-α in the colon of mice with DSS colitis, 1 and 2 mg/kg of the drug caused a similar reduction of more than 70% in TNF-α in the colon of rats with DNBS-induced colitis. Moreover, others have reported a reduction in colonic TNF-α by a cholinergic agonist anabaseine, in mice in which colitis was induced by DNBS [37]. Together with our findings, this indicates that the particular cytokine reduced by activation of the cholinergic system depends on the mode of induction of the colitis and not on the animal species.

In view of these data in two different rodent models, it is possible that rivastigmine can provide significant amelioration of the symptoms of colitis in human subjects. It is desirable that treatment should be administered via the skin patch rather than the conventional oral medication as this considerably reduces the adverse cholinergic effects of the drug.

### Summary

We have provided experimental evidence that rivastigmine (1–2 mg/kg) can significantly reduce overt signs and the accompanying structural and inflammatory changes of acute colitis induced in two experimental models in mice and rats which represent the pathophysiological features of UC and CD. ChE inhibition of 57–67% in macrophages, cortex, brainstem, colon and plasma is necessary to achieve the optimal anti-inflammatory activity without adverse effects. Rivastigmine has some advantages over selective new and existing nicotinic agonists for treating colitis. Its safety profile has been established in humans and it can act at different sites to potentiate the effect of ACh on both muscarinic and nicotinic receptors. These sites include M1 receptors in the brainstem, nAChR in vagal and splenic nerve ganglia and α7-nAChR on circulating macrophages. It remains to be seen if the cholinergic anti-inflammatory system can play a protective role in UC in humans and whether rivastigmine can provide any benefit in this condition.
